# The Use of Wearable Pulse Oximeters in the Prompt Detection of Hypoxemia and During Movement: Diagnostic Accuracy Study

**DOI:** 10.2196/28890

**Published:** 2022-02-15

**Authors:** Mauro Santos, Sarah Vollam, Marco AF Pimentel, Carlos Areia, Louise Young, Cristian Roman, Jody Ede, Philippa Piper, Elizabeth King, Mirae Harford, Akshay Shah, Owen Gustafson, Lionel Tarassenko, Peter Watkinson

**Affiliations:** 1 Institute of Biomedical Engineering Department of Engineering Science University of Oxford Oxford United Kingdom; 2 National Institute for Health Research Oxford Biomedical Research Centre Oxford United Kingdom; 3 Critical Care Research Group Nuffield Department of Clinical Neurosciences University of Oxford Oxford United Kingdom; 4 Adult Intensive Care Unit Oxford University Hospitals National Health Service Foundation Trust Oxford United Kingdom; 5 Therapies Clinical Service Oxford University Hospitals National Health Service Foundation Trust Oxford United Kingdom; 6 Radcliffe Department of Medicine University of Oxford Oxford United Kingdom

**Keywords:** diagnostic accuracy, hypoxia, hypoxemia, wearable pulse oximeter, continuous monitoring, mHealth, wearable technology, patient monitoring, deterioration, blood oxygen, hospital

## Abstract

**Background:**

Commercially available wearable (ambulatory) pulse oximeters have been recommended as a method for managing patients at risk of physiological deterioration, such as active patients with COVID-19 disease receiving care in hospital isolation rooms; however, their reliability in usual hospital settings is not known.

**Objective:**

We report the performance of wearable pulse oximeters in a simulated clinical setting when challenged by motion and low levels of arterial blood oxygen saturation (SaO_2_).

**Methods:**

The performance of 1 wrist-worn (Wavelet) and 3 finger-worn (CheckMe O2+, AP-20, and WristOx2 3150) wearable, wireless transmission–mode pulse oximeters was evaluated. For this, 7 motion tasks were performed: at rest, sit-to-stand, tapping, rubbing, drinking, turning pages, and using a tablet. Hypoxia exposure followed, in which inspired gases were adjusted to achieve decreasing SaO_2_ levels at 100%, 95%, 90%, 87%, 85%, 83%, and 80%. Peripheral oxygen saturation (SpO_2_) estimates were compared with simultaneous SaO_2_ samples to calculate the root-mean-square error (RMSE). The area under the receiver operating characteristic curve was used to analyze the detection of hypoxemia (ie, SaO_2_<90%).

**Results:**

SpO_2_ estimates matching 215 SaO_2_ samples in both study phases, from 33 participants, were analyzed. Tapping, rubbing, turning pages, and using a tablet degraded SpO_2_ estimation (RMSE>4% for at least 1 device). All finger-worn pulse oximeters detected hypoxemia, with an overall sensitivity of ≥0.87 and specificity of ≥0.80, comparable to that of the Philips MX450 pulse oximeter.

**Conclusions:**

The SpO_2_ accuracy of wearable finger-worn pulse oximeters was within that required by the International Organization for Standardization guidelines. Performance was degraded by motion, but all pulse oximeters could detect hypoxemia. Our findings support the use of wearable, wireless transmission–mode pulse oximeters to detect the onset of clinical deterioration in hospital settings.

**Trial Registration:**

ISRCTN Registry 61535692; http://www.isrctn.com/ISRCTN61535692

**International Registered Report Identifier (IRRID):**

RR2-10.1136/bmjopen-2019-034404

## Introduction

Failure to recognize and act on physiological indicators of worsening acute illness in hospital wards is a prevalent problem first recognized over 20 years ago [1-3]. Current practice involves intermittent measurements of vital signs and use of early warning scores [4], which are limited by the intermittent nature of the measurements and the associated time burden for staff [5]. Monitoring vital signs continuously with wearable (ambulatory) devices may overcome these limitations and improve detection of deterioration [6-8]. However, recent pilot and observational studies of wearable monitoring devices have shown mixed results, and no large clinical trials of ambulatory monitoring systems (AMSs) have demonstrated improved patient outcomes [6,9,10]. For example, in pulse oximetry, it is well known that patient motion and low perfusion in extremities can generate artifacts that reduce the accuracy of peripheral oxygen saturation (SpO_2_) readings [11]. This represents a major barrier to the deployment of these wearable devices for in-hospital patient monitoring [12]. Data averaging, alarm delay, and data holding are some of the strategies developed by pulse oximeter manufacturers to reduce the effect of motion artifacts and avoid false alerts [13], but there is still a need for studies of diagnostic accuracy and motion artifacts to support development of reliable wearable devices [3,7,14,15]. This need has become acute as health care systems have recommended the incorporation of ambulatory pulse oximeters in the home management of COVID-19 [16-18].

This study is part of a phased mixed-methods research project aiming to develop and refine an AMS using wearable devices to aid in the detection of deterioration and improve patient outcomes. The primary objective of this study was to determine the specificity and sensitivity of currently available ambulatory vital sign–monitoring equipment for the detection of hypoxemia. The secondary objective was to determine the effect of motion on data acquisition by the same devices.

## Methods

### Ethics

This research publication follows the Standards for Reporting Diagnostic Accuracy Studies reporting guidelines [19] and reports the results of the study protocol of Areia et al [20]. This study received ethics approval from the East of Scotland Research Ethics Service REC 2 (19/ES/0008) and was registered in June 2019 (no. ISRCTN61535692).

### Study Design

This was a prospective, observational study in which SpO_2_ estimates from the study devices were compared with the gold-standard arterial blood oxygen saturation (SaO_2_) samples and clinical-standard SpO_2_ estimates collected from arterial blood gas (ABG) samples and a nonambulatory Philips MX450 (Philips, Amsterdam, the Netherlands) pulse oximeter, respectively. The device’s pulse rate estimation accuracy is reported in Multimedia Appendix 1.

### Participants

Healthy adults (18 years or older) able to give informed consent for participation in the study were recruited consecutively from the Oxford area (United Kingdom) between June 18 and August 8, 2019. The exclusion criteria are described in detail in the study protocol [20], including clinical conditions that might bias the estimation of SpO_2_ by oximetry (eg, anemia) or increase risk to the participants’ health (eg, clotting disorders).

### Test Methods

#### Study Sessions

The study sessions took place at the Cardiovascular Clinical Research Facility, John Radcliffe Hospital, Oxford, UK. An arterial line was first inserted, under local anesthesia, preferentially into the nondominant radial artery of participants placed in the semirecumbent position (30^o^ head up). Where it was not possible to cannulate the nondominant arm, the dominant arm was cannulated. Participants wore 1 wrist-only device (Wavelet; WaveletHealth, Mountain View, USA) and 3 wrist-worn pulse oximeters with a finger probe: CheckMe O2+ (Viatom Technology Co Ltd, Shenzhen, China), AP-20 (Shenzhen Creative Industry Co Ltd, Shenzhen, China), and WristOx2 3150 with Bluetooth Low Energy (BLE; Nonin Medical Inc, Plymouth, USA) on the same arm. These devices are among the few that make both numeric and waveform data available to other systems. This is a requirement in our research [20] as we plan to notify clinical staff about the signal quality of the waveforms from which the numeric estimates are derived. A nonambulatory Philips MX450 pulse oximeter was also worn. CheckMe O2+ was always placed on the first finger as per the manufacturer’s recommendation. The position of the other 3 finger probes on the second, third, and fourth fingers was randomized using software from Haahr [21], per study visit day, ensuring an even distribution of placement. The participants also wore a 3-lead electrocardiogram (ECG) and an end-tidal carbon dioxide monitor connected to the Philips MX450 monitor, and an adhesive chest patch, for monitoring and acquisition of the heart rate and breathing rate. Results obtained with the chest patch are not reported here.

#### Stage 1: Movement Phase

An at-rest window was assigned to the period before the first ABG measurement, taken after fitting all the devices. The participants then moved to a chair and were asked to complete a series of consecutive motion tasks: 20 times sit-to-stand (STS), 2-minute tapping at 2 Hz, 2-minute rubbing at 2 Hz, 20 times drinking from a plastic cup, 50 times turning pages, and a set of predefined tablet activity tasks [20]. ABG measurements were made at the end of each motion task in order to analyze the mean bias of the SpO_2_ estimates for each task. For a sample of 15 participants, an additional ABG measurement was made in the middle of the STS motion to assess differences in the SaO_2_ during and after that activity.

#### Stage 2: Hypoxia Exposure Phase

Participants moved to a semirecumbent, supine position and wore a tight-fitting silicone facemask connected to a device that reduces the inspired fraction of oxygen, the hypoxicator unit (Everest Summit Hypoxic Generator, Altitude Centre, London, UK). During this phase, oxygen saturation from the clinical-standard Philips MX450 monitor guided the titration of the hypoxicator by a senior anesthetist from the research team, with appropriate resuscitation facilities nearby. In addition, 7% oxygen in nitrogen was used to further lower the fraction of inspired oxygen (FiO_2_), if required [22]. FiO_2_ was also monitored via an in-line gas analyzer. ABGs were sampled when the participants reached stable prespecified target peripheral oxygen saturation levels: 95%, 90%, 87%, 85%, 83%, and 80%. A senior anesthetist decided when a stable oxygen level was achieved in order to take the ABG based on the clinical values shown by the standard SpO_2_ monitor.

### Data Collection

Demographic data, including age, sex, height, weight, skin type (Fitzpatrick scale [23]), baseline heart rate (Philips MX450 3-lead ECG), and SaO_2_ (from the initial ABG), were collected for each participant at the start of their session. All data collection devices and software were synchronized to the same timestamp, at the start of each study session. SpO_2_ data (1 Hz) from CheckMe O2+ and WristOx2 3150 were sent via BLE to and timestamped in different Android tablets (application developed in-house). The AP-20 SpO_2_ data (1 Hz) were captured in the device and then downloaded via Oximeter Data Manager version 5.6 software (Shenzhen Creative Industry Co. Ltd., China). The Wavelet device first uploaded the photoplethysmography data to its web platform via an iOS app “On-site.” The platform then retrospectively estimated SpO_2_ (1 Hz), and these data were shared with the research team. The Phillips MX450 SpO_2_ data were collected using ixTrend version 2.1 software. The start and stop times of each motion task and the ABG measurement timings were recorded in case report forms. Functional SaO_2_ values were determined immediately after each ABG sample was taken, by multiwavelength oximetry, using a calibrated blood gas electrolyte analyzer, Radiometer ABL90 Flex (Radiometer, Copenhagen, Denmark).

### Statistical Analysis

#### Sample Size

The sample size calculation was based on the International Organization for Standardization (ISO) 80601-2-61:2019 guideline for testing the accuracy of pulse oximeters, which requires at least 200 data points balanced across the SaO_2_ range of 70%-100% from at least 10 subjects. We aimed to collect approximately 30 full data sets (with 7 ABGs being used in both the movement and hypoxia exposure phases, yielding a total of 420 readings, ie, 210 for each phase) to achieve a sufficient number of data points for the primary and secondary outcomes, and to recruit participants varying in their physical characteristics to the greatest extent possible. We excluded participants if incomplete data were collected for any 1 device during testing or if hypoxia was not achieved.

#### Accuracy, Bias, and Precision Metrics

Demographics and baseline vital sign descriptors were summarized using the mean, the median, and the first and third quartiles for continuous variables and proportions for categorical variables. In accordance with the ISO guideline, the accuracy of the SpO_2_ estimates for each device was determined using the root-mean-square error (RMSE) between the measured values (SpO_2i_) and the reference values (SaO_2i_):




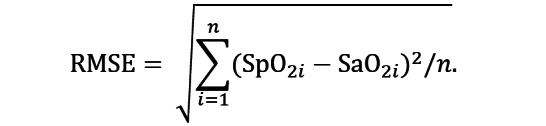




The RMSE 95% CI was determined using bootstrapping (random sampling with replacement) with 10,000 repetitions. The ISO guideline requires that valid oximeters present an RMSE below or equal to 4% (and below or equal to 8% when considering the CI). To interpret potential sources of the SpO_2_ estimation error, the mean bias B and precision S were also calculated as




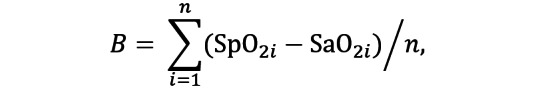




and




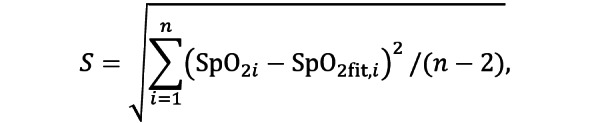




respectively. The latter is also known as the SD of the residuals, which determines the spread of the test SpO_2_ data around the linear regression model, SpO_2fit_, which predicts the SpO_2_ estimates that best fit the reference SaO_2_ values. The agreement between the test devices and the gold standard was also assessed via Bland-Altman plots. Finally, the mean absolute bias was also analyzed.

#### Movement Phase

The metrics were computed using the median SpO_2i_ from the 40-second window immediately before the stop time from each motion task and the SaO_2_ value from the ABG taken immediately after the same motion task.

#### Hypoxia Exposure Phase

The metrics were computed using the median SpO_2i_ from a 40-second window, including 35 seconds before and 5 seconds after the i-th reference SaO_2_ value (note that SaO_2_ readings were taken for the 80%, 83%, 85%, 87%, 90%, 95%, and 100% target values, with the corresponding output of the blood gas analyzer then taken as the reference value). These metrics were also computed for 3 SaO_2_ subgroups: severe hypoxia, SaO_2_ lower than 85%; mild hypoxia, SaO_2_ from 85% to 89%; and normoxia, SaO_2_ equal to or greater than 90%.

#### Statistical Tests

For both phases, one-way ANOVA followed by the Tukey-Kramer test [24] was used to evaluate differences in the mean bias and the mean absolute bias between groups. The Levene test [25] was used to evaluate differences in the precision between groups. In the movement phase, the distributions between the 15 additional SaO_2_ values taken at the middle of the STS motion and those taken at the end were compared via the Wilcoxon test. Significance was considered at *P*<.05.

#### Sensitivity and Specificity in Detecting Hypoxemia

To evaluate each device’s diagnostic accuracy in detecting hypoxemia, we determined the sensitivity, specificity, positive and negative predictive values (PPV and NPV), and accuracy (computed from the error matrix) for identifying values of SaO_2_ below 90%. To consider whether device performance would be more reliable if recalibrated, we calculated the area under the receiver operating characteristic (AUROC) curve for each pulse oximeter and computed the same metrics at the optimal operating value. In addition, 95% CIs for all metrics were determined using bootstrapping.

Due to SpO_2_ estimation performance issues, Wavelet analysis was removed. Its results can be found in Multimedia Appendix 2.

## Results

### Participants

**Figure 1:**
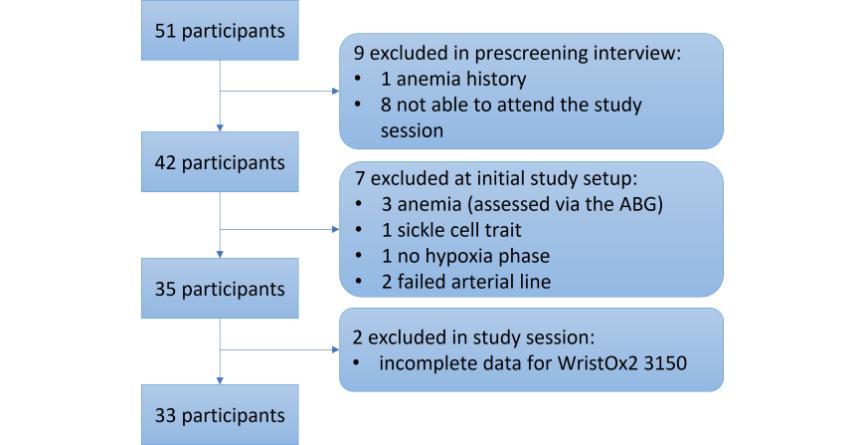
CONSORT flow diagram of the study. ABG: arterial blood gas; CONSORT: Consolidated Standards of Reporting Trials.

**Figure 2:**
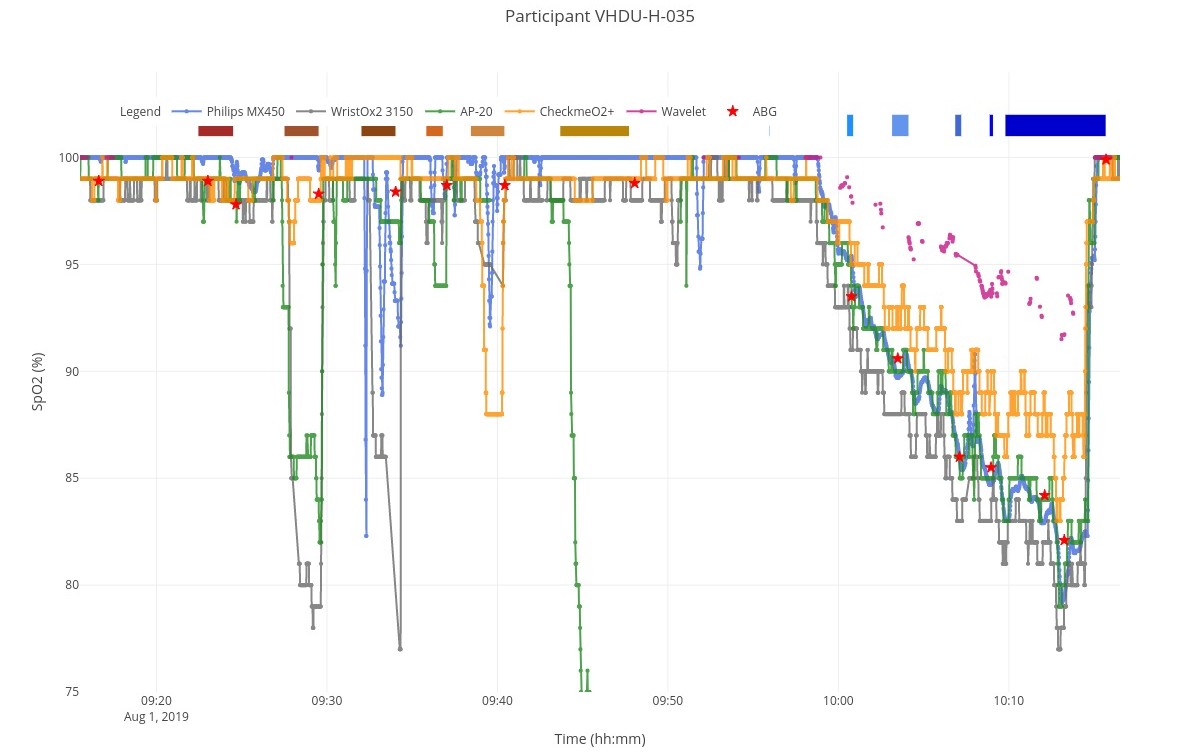
SpO_2_ trend for each device during the movement (9:20 AM-9:50 AM) and hypoxia exposure (9:58 AM-10:14 AM) phases of 1 study session. The gold-standard SaO_2_, derived from ABG samples, are shown as red stars. The different motion tasks and target desaturation intervals are illustrated by brown and blue rectangles at the top, respectively. Wavelet SpO_2_ data are shown for comparison (results can be reviewed in Multimedia Appendix 2). ABG: arterial blood gas; SaO_2_: arterial blood oxygen saturation; SpO_2_: peripheral oxygen saturation.

Prescreening interviews were performed on 51 volunteers (Consolidated Standards of Reporting Trials [CONSORT] flow diagram in Figure 1). Of these, 1 (2%) volunteer was excluded due to a history of anemia, and 8 (16%) were not able to attend the study session. The remaining 42 (82%) participants attended a study session: 4 (10%) participants presented clinical conditions, evaluated at the start of their session, that were part of the exclusion criteria and would bias the SpO_2_ estimations if included (3 [75%] additional anemia cases, evaluated from the first ABG, and 1 [25%] sickle cell trait); for 1 (2%) subject, it was not possible to induce hypoxia; for 2 (5%) subjects, it was not possible to insert an arterial line (in either arm); finally, 2 (5%) subjects had incomplete SpO_2_ data for the WristOx2 3150 device. Complete data were therefore obtained from 33 (79%) healthy adults, 18 (55%) women and 15 (45%) men, spread across Fitzpatrick skin types 1-4, with a median age of 29 (SD 24-36) years. The baseline vital sign mean values, measured at the start of each session, were 71 beats per minute (bpm), 15 respirations per minute (rpm), 100% SaO_2_, 130/75 mmHg, and a derived BMI of 23.7 kg/m^2^ (demographics available in Table 1). Figure 2 shows an exemplar SpO_2_ trend for each device for 1 study session. In general, poor estimation performance could be seen during the motion tasks (identified by the brown periods at the top), and all devices (expect Wavelet, whose analysis can be reviewed in Multimedia Appendix 2) followed the SaO_2_ desaturation trend (red stars) during the hypoxia phase (from 9:58 AM to 10:14 AM). We discuss their accuracy next.

**Table 1:** Demographics and baseline heart rate, respiration rate, blood pressure, and SaO_2_^a^ for 33 participants.

Demographics		Mean (median)		Q1^b^, Q3^c^
Age (years)		29.0 (31.18)		24.0, 36.0
Sex (female), %		18 (54.5)		N/A^d^
Height (m)		1.70 (1.70)		1.6, 1.8
Weight (kg)		70.0 (70.7)		61.0, 80.0
BMI (kg/m^2^)		23.7 (24.3)		21.5, 26.4
**Skin tone^e^, %**				
	Type 1	9 (27.3)	N/A	
	Type 2	15 (45.5)	N/A	
	Type 3	2 (6.1)	N/A	
	Type 4	7 (21.2)	N/A	
	Type 5	0 (0)	N/A	
	Type 6	0 (0)	N/A	
Respiration rate (rpm^f^)		15.0 (15.7)		13.0, 18.0
Heart rate (bpm^g^)		71.0 (70.9)		62.0, 82.0
SaO_2_, %		100.0 (99.6)		100.0, 100.0
Systolic blood pressure (mmHg)		129.5 (133.8)		122.8, 142.8
Diastolic blood pressure (mmHg)		75.0 (77.4)		69.8, 86.3

^a^SaO_2_: arterial blood oxygen saturation.

^b^Q_1_: first quartile.

^c^ Q_3_: third quartile.

^d^N/A: not applicable.

^e^Fitzpatrick scale.

^f^rpm: respirations per minute.

^g^bpm: beats per minute.

### SpO_2_ Estimation in the Movement Phase

**Figure 3:**
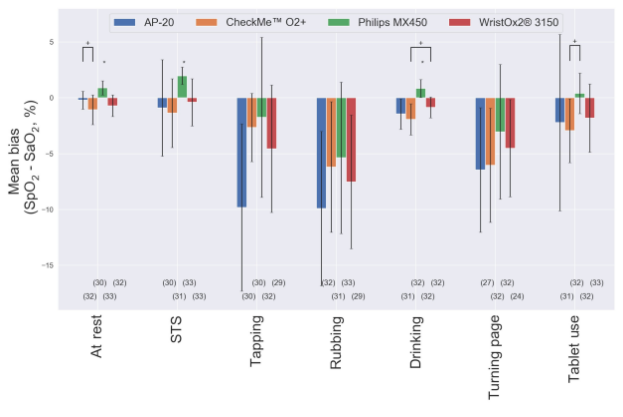
Comparison of the mean bias (SpO_2_–SaO_2_) and precision between devices for each movement type. The number of points available per device is presented below each bar. For each task, one-way ANOVA followed by the Tukey test was used to evaluate differences in the mean bias between devices. *Different from other values. +Different from each other. SaO_2_: arterial blood oxygen saturation; SpO_2_: peripheral oxygen saturation; STS: sit-to-stand.

The results of SpO_2_ estimation performance metrics for each device are shown in Table 2. The mean bias and precision are further illustrated for each motion task in Figure 3. The number of dropout values was comparable between the finger-worn devices. The RMSE values were below 4% when at rest and for the STS and drinking tasks for all devices. For all other tasks, they were above 4% for at least 1 device.

**Table 2:** Comparison of accuracy (RMSE^a^) and bias in SpO_2_^b^ estimation between different motion tasks, for each device, for 33 participants.

Performance metrics		At rest	STS^c^	Rubbing	Tapping	Drinking	Turning pages	Tablet use	*P* value^d^
**AP20**									
	Available SpO_2_ points, n	32	30	32	30	31	27	31	N/A^e^
	RMSE, % (95% CI)	0.82 (0.55-1.06)	4.68 (1.47-7.72)	11.96 (9.44-14.23)	12.21 (9.31-14.74)	1.96 (1.48-2.46)	8.52 (6.18-10.75)	8.01 (1.15-13.72)	N/A
	Mean bias, %	–0.21^f,g^	–0.9^h,i^	–9.91^f,g,j,k^	–9.82^g,i^	–1.45^j^	–6.46	–2.22^k^	<.001
	Mean |bias|, %	0.6^k,l,m^	2.15^f,g^	9.91^f,i,j,k^	9.85^g,h,l,n^	1.57^h,j^	6.46^m^	2.56^i,n^	<.001
	Precision, %	0.81^f^	4.31^h^	6.91	7.49^f,h,j,k^	1.37^j^	5.57	7.89^k^	<.001
**CheckMe** **O2+**									
	Available SpO_2_ points, n	30	31	31	30	32	32	32	N/A
	RMSE, % (95% CI)	1.68 (1.21-2.12)	3.5 (1.49-5.37)	8.45 (5.86-10.88)	3.99 (2.28-5.69)	2.43 (1.9-2.96)	7.83 (5.9-9.8)	4.2 (2.86-5.47)	N/A
	Mean bias, %	–1.06	–1.37	–6.19^j^	–2.65^h,j^	–1.93	–6.04^h^	–2.94	.001
	Mean |bias|, %	1.29	1.92	6.31	2.71	1.97	6.06	2.98	.005
	Precision, %	1.33	3.08	5.84	3.06	1.41	5.11	2.86	.13
**Philips MX450**									
	Available SpO_2_ points, n	33	33	33	32	32	32	32	N/A
	RMSE, % (95% CI)	1.11 (0.92-1.28)	2.31 (1.9-2.67)	9.49 (7.04-11.86)	7.15 (3.07-10.3)	1.17 (1.0-1.36)	6.64 (3.81-9.03)	1.97 (1.29-2.68)	N/A
	Mean bias, %	0.89^f^	1.97^h^	–5.37^f,h,i,j,k^	–1.75^i^	0.84^j^	–3.04	0.4^k^	<.001
	Mean |bias|, %	0.97^h^	2.02	6.6^h,j^	3.33	1.06^j^	4.03	1.51	.002
	Precision, %	0.63^g,k^	0.78^f^	6.77^f,i,j,k^	7.16^g,h^	0.8^h,j^	6.04	1.82^i^	<.001
**WristOx2 3150**									
	Available SpO_2_ points, n	32	33	29	29	32	24	33	N/A
	RMSE, % (95% CI)	1.18 (0.84-1.51)	2.33 (1.26-3.41)	9.5 (7.29-11.5)	7.17 (4.66-9.35)	1.27 (0.95-1.57)	6.28 (4.25-8.27)	3.91 (1.49-5.62)	N/A
	Mean bias, %	–0.71^f^	–0.4^h^	–7.52^f,h,i,j,k^	–4.56^i^	–0.86^j^	–4.51	–1.81^k^	.002
	Mean |bias|, %	0.92^k,l^	1.38^f,g^	7.52^f,i,j,k^	4.69^g,l,m^	1.02^h,j^	4.56	2.02^h,m^	<.001
	Precision, %	0.97^f^	2.12^h^	5.98	5.7^f,h,j,k^	0.93^j^	4.35	3.06^k^	.001

^a^RMSE: root-mean-square error.

^b^SpO_2_: peripheral oxygen saturation.

^c^STS: sit-to-stand.

^d^One-way ANOVA followed by the Tukey-Kramer test was used to evaluate differences in the mean bias and mean absolute bias between tasks. The Levene test was used in the case of precision.

^e^N/A: not applicable.

^f-n^Different from each other; for example, for CheckMe O2+, the mean bias of the tapping motion task was different from that of the turning page task and that of the rubbing task (paired differences coded as ^j^ and ^h^, respectively).

### SpO_2_ Estimation in the Hypoxia Exposure Phase

**Figure 4:**
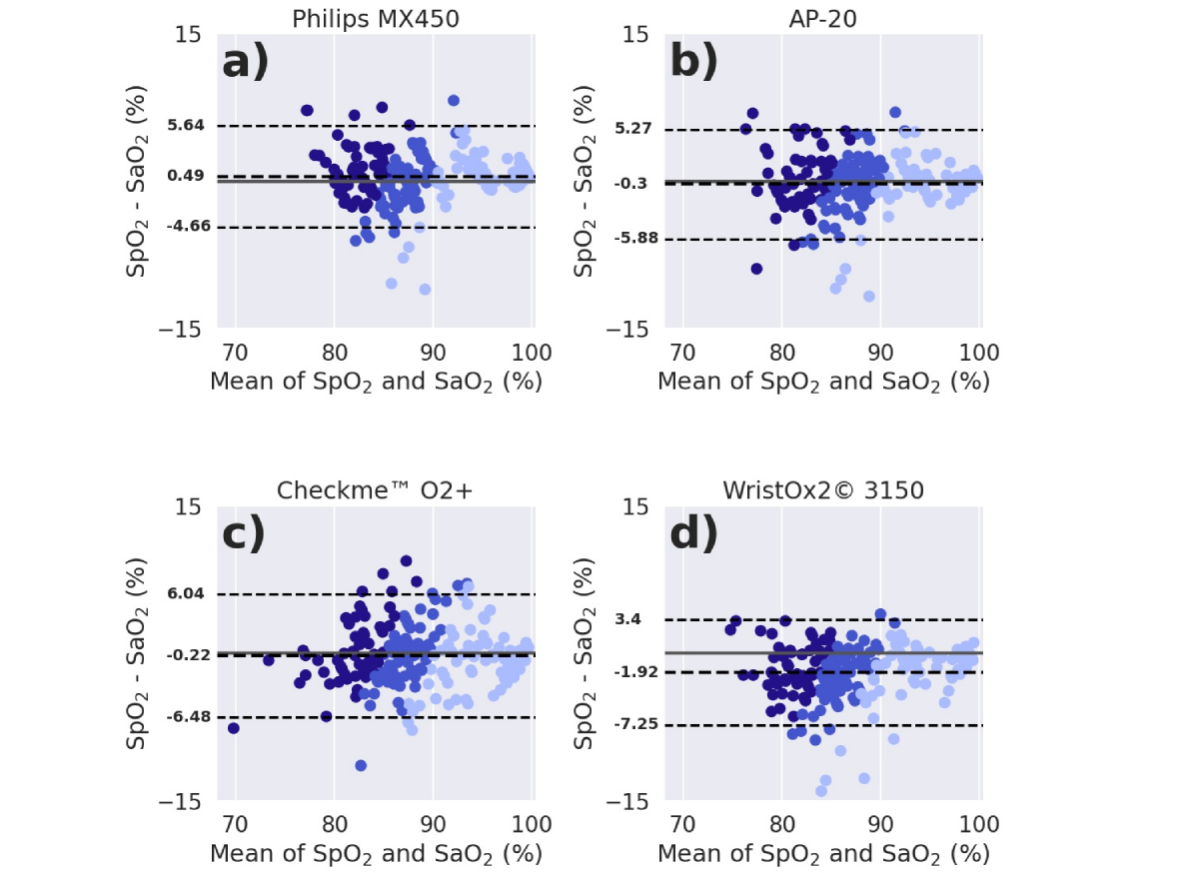
(a-d) Bland-Altman plots for the Philips MX450, AP-20, CheckMe O2+, and WristOx2 3150 SpO_2_ estimation, respectively. The mean bias and limits of agreement values are shown at the left of their respective dashed lines. The solid line represents y=0 (no bias). SaO_2_: arterial blood oxygen saturation; SpO_2_: peripheral oxygen saturation.

**Figure 5:**
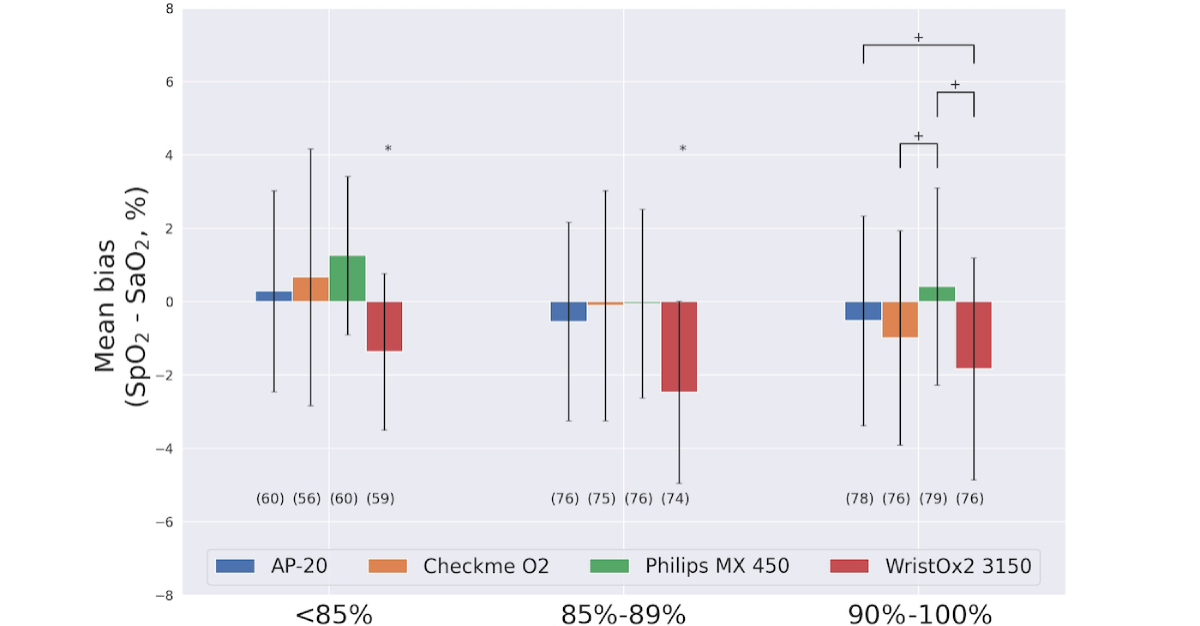
Comparison of the mean bias (SpO_2_–SaO_2_) and precision between devices for the 3 SaO_2_ subgroups: severe hypoxia, SaO_2_<85%; mild hypoxia, SaO_2_=85%-89%; and normoxia, SaO_2_=90%-100%. The number of points available per device is presented below each bar. For each subgroup, one-way ANOVA followed by the Tukey test was used to evaluate differences in the mean bias between devices. *Different from other values. +Different from each other. SaO_2_: arterial blood oxygen saturation; SpO_2_: peripheral oxygen saturation.

Table 3 compares the SpO_2_ estimation performance of the devices across the range of SaO_2_ targets of the hypoxia exposure phase, and their Bland-Altman plots (with the mean bias and limits of agreement) can be reviewed in Figure 4. The WristOx2 3150 device underestimated SpO_2_ in comparison with SaO_2_ by almost 2%. The WristOx2 3150 and CheckMe O2+ devices had the numerically greatest mean absolute bias. However, SaO_2_ subgroup analysis (see Figure 5 and Table 4) showed that the WristOx2 3150 device consistently underestimated SpO_2_ across the measured range (with an overall mean bias of –1.92% [SD 2.73%]; Table 3), whereas the CheckMe O2+ device overestimated in the severe-hypoxia range and underestimated in the mild-hypoxia and normoxia ranges (Figure 5). However, WristOx2 3150, CheckMe O2+, AP-20, and Philips MX450 showed an overall RMSE below 4% (and below 8% when considering the 95% CI; Table 3), meeting the ISO 80601-2-61:2019 requirement.

**Table 3:** Comparison of accuracy (RMSE^a^) and mean bias of SpO_2_^b^ estimation between devices during the hypoxia exposure phase. There were 215 SaO_2_ target windows in this phase.

Performance metrics	Philips MX450	CheckMe O2+	WristOx2 3150	AP-20	*P* value
Available SpO_2_ points, n	215	207	209	214	N/A^c^
RMSE, % (95% CI)	2.67 (2.31-3.06)	3.20 (2.85-3.56)	3.33 (2.85-3.86)	2.86 (2.44-3.25)	N/A
Mean bias, %	0.49^d^	–0.22	–1.92^e^	–0.3^d^	<.001
Mean |bias|, %	1.92	2.42	2.40	2.00	<.02
Precision, %	2.62^d^	3.16^d^	2.73	2.83	<.02

^a^RMSE: root-mean-square error.

^b^SpO_2_: peripheral oxygen saturation.

^c^N/A: not applicable.

^d^Different from each other.

^e^Different from other values.

**Table 4:** Comparison of accuracy (RMSE^a^) and mean bias of the device’s SpO_2_^b^ estimation between 3 SaO_2_^c^ subgroups: severe hypoxia (SaO_2_<85%), mild hypoxia (SaO_2_ 85%-89%), and normoxia (SaO_2_≥90%).

Performance metrics			<85%		85%-89%		90%-100%		*P* value^d^
**Total ABGs^e^, n**			60		76		79		N/A^f^
**AP-20**									
	Available SpO_2_ points, n	60		76		78		N/A	
	RMSE*,* % (95% CI)	2.99 (2.44-3.57)		2.73 (2.28-3.16)		2.88 (1.88-3.78)		N/A	
	Mean bias*,* %	0.28		–0.54		–0.52		.18	
	Mean |bias|*,* %	2.33		2.07		1.68		.17	
	Precision*,* %	2.74		2.71		2.86		.16	
**CheckMe** **O2+**									
	Available SpO_2_ points, n	56		75		76		N/A	
	RMSE*,* % (95% CI)	3.52 (2.86-4.18)		3.10 (2.46-3.83)		3.05 (2.54-3.53)		N/A	
	Mean bias*,* %	0.67^d^		–0.11^d^		–0.99		.01	
	Mean |bias|*,* %	2.74		2.35		2.26		.39	
	Precision*,* %	3.5		3.14		2.92		.28	
**Philips MX 450**									
	Available SpO_2_ points, n	60		76		79		N/A	
	RMSE*,* % (95% CI)	2.80 (2.18-3.33)		2.54 (2.08-3.02)		2.70 (1.88-3.56)		N/A	
	Mean bias*,* %	1.26		–0.05^g^		0.42^g^		.02	
	Mean |bias|*,* %	2.13		1.97		1.72		.44	
	Precision*,* %	2.16		2.57		2.69		.24	
**WristOx2 3150**									
	Available SpO_2_ points, n	59		74		76		N/A	
	RMSE*,* % (95% CI)	2.69 (2.28-3.08)		3.49 (2.92-3.99)		3.61 (2.37-4.64)		N/A	
	Mean bias*,* %	–1.36		–2.47		–1.83		.06	
	Mean |bias|*,* %	2.21		2.83		2.12		.13	
	Precision*,* %	2.13		2.48		3.03		.99	

^a^RMSE: root-mean-square error.

^b^SpO_2_: peripheral oxygen saturation.

^c^SaO_2_: arterial blood oxygen saturation.

^d^For each device, one-way ANOVA followed by Tukey test was used to evaluate differences in the mean bias and mean absolute bias between subgroups. The Levene test was used in the case of precision.

^e^ABG: arterial blood gas.

^f^N/A: not applicable.

^g^Different from each other.

### Sensitivity and Specificity

**Figure 6:**
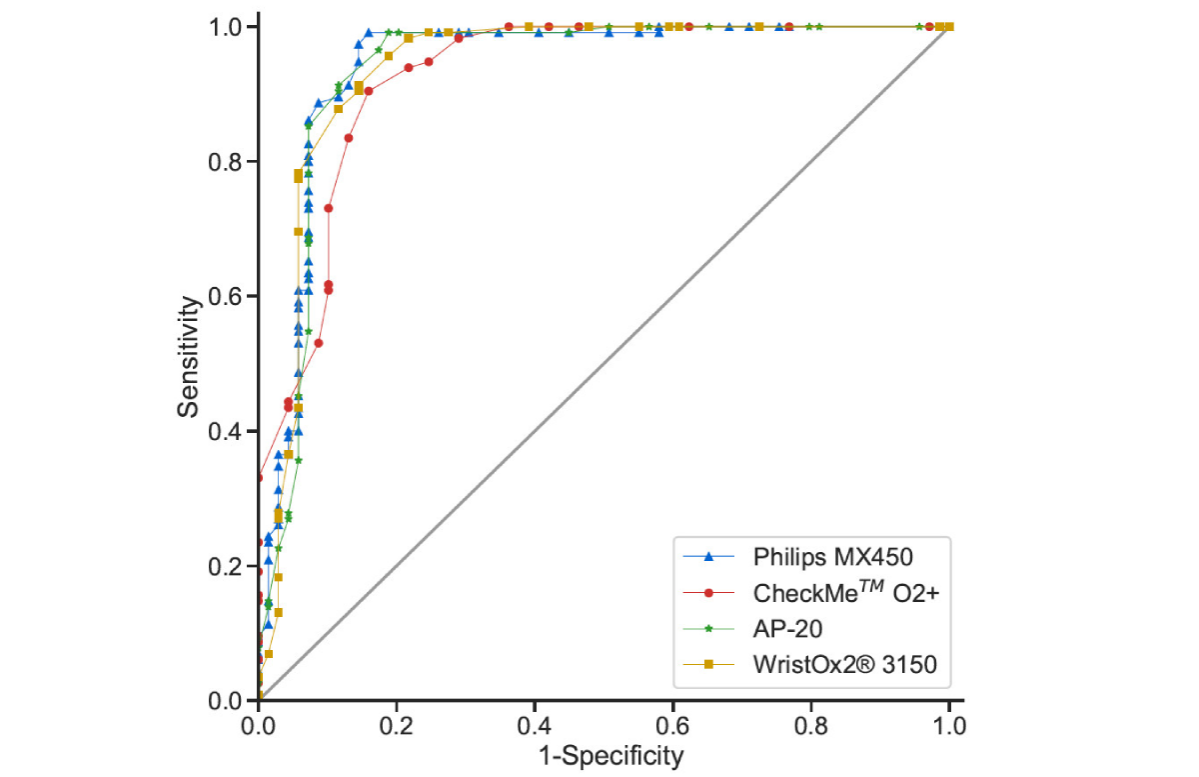
ROC curves in detecting hypoxemia (SaO_2_<90%) during the hypoxia exposure phase. ROC: area under the receiver operating characteristic; SaO_2_: arterial blood oxygen saturation.

Table 5 shows the performance metrics of the pulse oximeters in detecting hypoxemia (SaO_2_<90%; AUROC curves available in Figure 6). A total of 128 SaO_2_ targets were in the hypoxemia range versus 74 in the normoxia range. At a 90% cut-off, WristOx2 3150 showed significantly better sensitivity (0.97, 95% CI 0.93-0.99) than Philips MX450 (0.86, 95% CI 0.80-0.92). The values for the other metrics were comparable between all devices. All finger-worn devices achieved a good and comparable AUROC curve (≥0.92). Recalibration of the SpO_2_ threshold to the optimal operating value resulted in AP-20 achieving significantly higher sensitivity than CheckMe O2+ (0.95 [95% CI 0.91-0.98] vs 0.78 [95% CI 0.71-0.85]). The remaining sensitivity and specificity values were comparable.

**Table 5:** Performance metrics of each pulse oximeter for detecting hypoxemia (SaO_2_^a^<90%). The metrics are shown at a 90% SpO_2_^b^ cut-off and for the determined optimal SpO_2_ cut-off.

Device		Cut-off, %		AUROC^c^, mean (95% CI)	Sensitivity, mean (95% CI)		Specificity, mean (95% CI)		PPV^d^, mean (95% CI)		NPV^e^, mean (95% CI)		Accuracy^f^, mean (95% CI)
**90% SpO_2_ (%) cut-off**													
	Philips MX 450	90.0	N/A^g^		0.86 (0.80-0.92)	0.93 (0.87-0.99)		0.96 (0.92-0.99)		0.79 (0.71-0.88)		0.89 (0.84-0.93)	
	CheckMe O2+	90.0	N/A		0.87 (0.81-0.93)	0.85 (0.76-0.93)		0.91 (0.86-0.96)		0.80 (0.71-0.88)		0.87 (0.82-0.91)	
	WristOx2 3150	90.0	N/A		0.97 (0.93-0.99)	0.80 (0.70-0.89)		0.89 (0.84-0.94)		0.94 (0.87-0.99)		0.91 (0.86-0.95)	
	AP-20	90.0	N/A		0.91 (0.85-0.95)	0.89 (0.82-0.96)		0.94 (0.89-0.98)		0.85 (0.76-0.92)		0.90 (0.86-0.94)	
**Optimal SpO_2_ (%) cut-off obtained via AUROC analysis^h^**													
	Philips MX 450	90.7	0.94 (0.90-0.98)		0.97 (0.94-0.99)	0.86 (0.78-0.94)		0.93 (0.88-0.97)		0.94 (0.88-0.99)		0.93 (0.90-0.97)	
	CheckMe O2+	89.0	0.92 (0.87-96)		0.78 (0.71-0.85)	0.88 (0.80-0.95)		0.92 (0.86-0.97)		0.70 (0.60-0.79)		0.82 (0.76-0.87)	
	WristOx2 3150	88.0	0.94 (0.89-97)		0.88 (0.82-0.94)	0.86 (0.78-0.94)		0.92 (0.87-0.96)		0.81 (0.72-0.89)		0.88 (0.83-0.92)	
	AP-20	91.0	0.94 (0.89-98)		0.95 (0.91-0.98)	0.84 (0.75-0.92)		0.91 (0.86-0.96)		0.91 (0.84-0.97)		0.91 (0.87-0.95)	

^a^SaO_2_: arterial blood oxygen saturation.

^b^SpO_2_: peripheral oxygen saturation.

^c^AUROC: area under the receiver operating characteristic.

^d^PPV: positive predictive value.

^e^NPV: negative predictive value.

^f^Accuracy = (True positives + True negatives)/n, where n is the total number of examples.

^g^N/A: not applicable.

^h^The optimal SpO_2_ cut-off is the best compromise between sensitivity and specificity to detect hypoxemia (SaO_2_<90%).

## Discussion

### Principal Findings

Several studies have been published on both the usefulness and the potential issues of pulse oximetry in the clinical setting using nonambulatory devices. In this study, we compared the performance of wearable pulse oximeters and 1 nonambulatory pulse oximeter using gold-standard arterial blood samples drawn from healthy adult participants. Availability of waveform data was a requirement that limited the selection of devices. Our provision of waveform data will allow clinical staff to assess the reliability of the signal. However, a risk with all continuous-monitoring systems is that they increase the burden on clinical teams by providing excess data. Further work is required to determine the usefulness of these systems in clinical practice and how continuous-monitoring data should be summarized in the electronic patient record.

In tests of finger-based devices, WristOx2 3150 significantly underestimated SaO_2_ (mean bias –1.92% [SD 2.73%]; Table 3) when compared with the other wearables. Nevertheless, all finger-based probes showed a similar mean absolute bias (about 2%) and RMSE (about 3%). Overall, all finger-worn wearable pulse oximeters achieved good sensitivity (≥0.87) and specificity (≥0.80), comparable to the standard nonambulatory device, in detecting hypoxemia (Table 5). Given that WristOx2 3150 underestimates SaO_2_, it presented higher sensitivity (0.97, 95% CI 0.93-0.99) at the cost of a lower specificity value (0.80, 95% CI 0.70-0.89). This underestimation explains why recalibration by 2% achieves the optimal operating point. The remaining devices only required a change in the threshold by 1% at their optimal operating point.

From the 7 motion tasks, tapping, rubbing, turning book pages, and using a tablet challenged the finger-based wearable devices the most (the first 2 are also analyzed by Louie et al [11] and Barker and Shah [26]), resulting in an RMSE above 4% in at least 1 device. The mean bias at rest, STS, and drinking motions was comparable (<4%; Table 2).

### Limitations

The sample size calculation for our study was based on the ISO 80601-2-61:2019 guidelines to evaluate the accuracy of pulse oximeters in detecting changes in SpO_2_, not to identify differences in performance between pulse oximeters and between activities. The study was not designed to generalize results to the wider population, for example, for patients with darker skin types or with acute illness.

We chose to sample ABGs at the end of each task to avoid accidental removal of the cannula. However, it became clear during our study that the ABGs could have been sampled while the motion task was occurring, perhaps better representing that interval reference SaO_2_. Preliminary analysis of the difference between ABGs taken immediately after the STS motion task and those taken at the midpoint of that motion, for 15 patients, showed that the SaO_2_ dropped by an average of 1.87% (SD 0.87%, *P*<.001 between the 2 SaO_2_ sample sets), indicating that the SpO_2_ value might change between the time used to compute the SpO_2_ estimates and that of the ABG samples taken after the exercise. Our hypothesis was that the STS task would be the motion task with the greatest effect on the participants’ SaO_2_. However, the error in the SpO_2_ estimates from the wearable devices during motion was much larger, so this correction would not have changed our findings.

### Conclusion

The accuracy of SpO_2_ estimation by finger-worn pulse oximeters was within that required by the ISO 80601-2-61:2019 guideline (≤4%). The accuracy was degraded by motion but not more than that with usual-care bedside monitors. All finger-worn pulse oximeters were capable of detecting hypoxemia, their performance being comparable to that used in nonambulatory standard care.

Our findings support the use of wearable, finger-based, wireless transmission–mode pulse oximeters to detect the onset of clinical deterioration in the hospital, possibly earlier than intermittent vital-sign measurements. The continuous assessment of SpO_2_, especially values below 90%, may be helpful to manage the care of ambulatory in-hospital patients who have been infected with the SARS-CoV-2 virus [27]. Further work is required to assess the impact of AMSs on patient outcomes, both during the COVID-19 pandemic and beyond.

## Appendix

Multimedia Appendix 1 Analysis of the pulse rate estimation in wearable pulse oximeters.

Multimedia Appendix 2 Results of Wavelet’s SpO_2_ estimation.

## Abbreviations

ABG: arterial blood gas
AMS: ambulatory monitoring system
AUROC: area under the receiver operating characteristic
CONSORT: Consolidated Standards of Reporting Trials
BLE: Bluetooth Low Energy
bpm: beats per minute
ECG: electrocardiogram
FiO_2_: fraction of inspired oxygen
ISO: International Organization for Standardization
NPV: negative predictive value
PPV: positive predictive value
RMSE: root-mean-square error
ROC: receiver operating characteristic
rpm: respirations per minute
SaO_2_: arterial blood oxygen saturation
SpO_2_: peripheral oxygen saturation
STS: sit-to-stand

## References

[1] Sun L, Joshi M, Khan SN, Ashrafian H, Darzi A (2020). Clinical impact of multi-parameter continuous non-invasive monitoring in hospital wards: a systematic review and meta-analysis. J R Soc Med.

[2] McQuillan P, Pilkington S, Allan A, Taylor B, Short A, Morgan G, Nielsen M, Barrett D, Smith G, Collins CH (1998). Confidential inquiry into quality of care before admission to intensive care. BMJ.

[3] Leenen JPL, Leerentveld C, van Dijk JD, van Westreenen HL, Schoonhoven L, Patijn GA (2020). Current evidence for continuous vital signs monitoring by wearable wireless devices in hospitalized adults: systematic review. J Med Internet Res.

[4] Royal College of Physicians (2017). National Early Warning Score (NEWS) 2. Standardising the Assessment of Acute-Illness Severity in the NHS.

[5] Downey CL, Tahir W, Randell R, Brown JM, Jayne DG (2017). Strengths and limitations of early warning scores: a systematic review and narrative synthesis. Int J Nurs Stud.

[6] Downey C, Randell R, Brown J, Jayne DG (2018). Continuous versus intermittent vital signs monitoring using a wearable, wireless patch in patients admitted to surgical wards: pilot cluster randomized controlled trial. J Med Internet Res.

[7] Joshi M, Ashrafian H, Aufegger L, Khan S, Arora S, Cooke G, Darzi A (2019). Wearable sensors to improve detection of patient deterioration. Expert Rev Med Devices.

[8] Watkins T, Whisman L, Booker P (2016). Nursing assessment of continuous vital sign surveillance to improve patient safety on the medical/surgical unit. J Clin Nurs.

[9] Weller RS, Foard KL, Harwood TN (2018). Evaluation of a wireless, portable, wearable multi-parameter vital signs monitor in hospitalized neurological and neurosurgical patients. J Clin Monit Comput.

[10] Paul JE, Chong MA, Buckley N, Harsha P, Shanthanna H, Tidy A, Buckley D, Clarke A, Young C, Wong T, Vanniyasingam T, Thabane L (2019). Vital sign monitoring with continuous pulse oximetry and wireless clinical notification after surgery (the VIGILANCE pilot study): a randomized controlled pilot trial. Pilot Feasibility Stud.

[11] Louie A, Feiner J, Bickler P, Rhodes L, Bernstein M, Lucero J (2018). Four types of pulse oximeters accurately detect hypoxia during low perfusion and motion. Anesthesiology.

[12] Appelboom G, Camacho E, Abraham ME, Bruce SS, Dumont EL, Zacharia BE, D'Amico R, Slomian J, Reginster JY, Bruyère Olivier, Connolly ES (2014). Smart wearable body sensors for patient self-assessment and monitoring. Arch Public Health.

[13] Petterson MT, Begnoche VL, Graybeal JM (2007). The effect of motion on pulse oximetry and its clinical significance. Anesth Analg.

[14] Weenk M, van Goor H, Frietman B, Engelen LJ, van Laarhoven CJ, Smit J, Bredie SJ, van de Belt TH (2017). Continuous monitoring of vital signs using wearable devices on the general ward: pilot study. JMIR Mhealth Uhealth.

[15] Verrillo SC, Cvach M, Hudson KW, Winters BD (2019). Using continuous vital sign monitoring to detect early deterioration in adult postoperative inpatients. J Nurs Care Qual.

[16] Seshadri DR, Davies EV, Harlow ER, Hsu JJ, Knighton SC, Walker TA, Voos JE, Drummond CK (2020). Wearable sensors for COVID-19: a call to action to harness our digital infrastructure for remote patient monitoring and virtual assessments. Front Digit Health.

[17] O'Carroll O, MacCann R, O'Reilly A, Dunican EM, Feeney ER, Ryan S, Cotter A, Mallon PW, Keane MP, Butler MW, McCarthy C (2020). Remote monitoring of oxygen saturation in individuals with COVID-19 pneumonia. Eur Respir J.

[18] Greenhalgh T, Knight M, A'Court C, Buxton M, Husain L (2020). Management of post-acute covid-19 in primary care. BMJ.

[19] Cohen JF, Korevaar DA, Gatsonis CA, Glasziou PP, Hooft L, Moher D, Reitsma JB, de Vet HC, Bossuyt PM, STARD Group (2017). STARD for Abstracts: essential items for reporting diagnostic accuracy studies in journal or conference abstracts. BMJ.

[20] Areia C, Vollam S, Piper P, King E, Ede J, Young L, Santos M, Pimentel MAF, Roman C, Harford M, Shah A, Gustafson O, Rowland M, Tarassenko L, Watkinson PJ (2020). Protocol for a prospective, controlled, cross-sectional, diagnostic accuracy study to evaluate the specificity and sensitivity of ambulatory monitoring systems in the prompt detection of hypoxia and during movement. BMJ Open.

[21] Haahr M What's This Fuss about True Randomness?.

[22] Rowland MJ, Ezra M, Winkler A, Garry P, Lamb C, Kelly M, Okell TW, Westbrook J, Wise RG, Douaud G, Pattinson KT (2019). Calcium channel blockade with nimodipine reverses MRI evidence of cerebral oedema following acute hypoxia. J Cereb Blood Flow Metab.

[23] Fitzpatrick TB (1988). The validity and practicality of sun-reactive skin types I through VI. Arch Dermatol.

[24] Haynes W, Dubitzky W, Wolkenhauer O, Cho K-H, Yokota H (2013). Tukey’s test. Encyclopedia of Systems Biology.

[25] NIST/SEMATECH (2012). NIST/SEMATECH e-Handbook of Statistical Methods.

[26] Barker SJ, Shah NK (1996). Effects of motion on the performance of pulse oximeters in volunteers. Anesthesiology.

[27] Oxford University Hospitals New Wearable Technology to Monitor COVID-19 Patients.

