# Supplementary material for: The Use of Wearable Pulse Oximeters in the Prompt Detection of Hypoxemia and During Movement: Diagnostic Accuracy Study
Source: J Med Internet Res. 2022 Feb 15;24(2):e28890. doi: 10.2196/28890 (PMC8889481; doi:10.2196/28890)
Supplement: Multimedia Appendix 1 [file jmir_v24i2e28890_app1.pdf]

Appendix A

Analysis of the pulse rate estimation in wearable pulse oximeters

Non-ambulatory pulse oximeters, used in standard monitors in hospital, estimate both peripheral oxygen saturation (SpO_2) and Pulse Rate (PR). PR can be derived from changes in the pulsating blood flow that are reflected in the amplitude of the detected light signals (the near-infrared signal is traditionally used in PR estimation by pulse oximeters). In this sub-study, the accuracy of PR estimates in selected wearable pulse oximeters, during a set of movements and a hypoxia exposure period, is evaluated against a standard monitor.

Materials and Methods

PR data was collected from all the pulse oximeters (at 1 Hz), alongside the SpO_2 data collection described in the methods section of the main manuscript. Reference Heart Rate (HR) was obtained from a standard Philips MX450 monitor 3-lead ECG (at 1 Hz). Both the test PR and the reference HR estimates were computed using median estimates from the 40-second windows considered in the main manuscript: 40 seconds before the stop of a motion task (movement phase); 35 seconds before and 5 seconds after an arterial blood gas sample was taken (hypoxia exposure phase). The root mean squared error, RMSE (and CI), mean bias, mean absolute bias, precision, and Bland-Altman plots (defined also in the main manuscript), were used to assess PR estimation accuracy. One-way ANOVA followed by the Tukey-Kramer test was used to evaluate differences in the mean bias and mean absolute bias, between groups. Levene's test was used to evaluate differences in the precision between groups. Significance was considered at a $P < 0.05$. The acceptable limit for PR estimation RMSE is not defined in the ISO 80601-2-61:2019. We therefore used that defined in the ANSI/AAMI EC13:2002 guideline, i.e. a RMSE $\leq$ 5%.

Results

Participants

The participant demographics matched those analysed in the main manuscript (results section). There was a total of 227 and 215 target HR windows available in the movement and hypoxia exposure phases, respectively.

PR estimation in the movement phase

All devices showed a RMSE above 5% in at least one motion task, the sit-to-stand (STS) and rubbing tasks showing the highest errors (Table B.1). E.g., the AP-20 showed a significantly higher mean bias in these two tasks (of -13.38 $\pm$ 10.94 bpm and 24.58 $\pm$ 14.51 bpm, respectively) than the Philips MX450 and the WristOx2® 3150. Although the Wavelet showed a comparable bias (Figure A.1), it still presented a low number of available PR estimates in most motion tasks (ranging from 2 to 8 data points), and only 18 PR estimates out of 33 possible target HR windows at rest.

Table A.1 – Comparison of the accuracy and the bias of PR estimation between different motion tasks, for each pulse oximeter, for 33 participants. The total number of HR target windows were 227, displayed in the top row, per activity. N – number of available test PR points. [+]Different from other values. [a,b,c,d,e,f,g,h,i,j,k] Different from each other.

	At Rest	***STS***	***Rubbing***	***Tapping***	***Drinking***	***Turning page***	***Tablet***	***P***
HR targets (n)	33	32	33	33	33	31	32	
AP-20								
N	32	29	32	31	31	26	30	-
RMSE (%, 95% CI)	5.34(2.39, 7.79)	17.15(13.23, 20.77)	17.35(12.18, 21.96)	29.18(24.06, 33.57)	4.13(2.84, 5.36)	4.99(3.82, 6.02)	6.93(2.49, 11.19)	-
Mean bias (%)	-0.73	-13.38[+]	-1.33	24.19[+]	-1.63	-2.12	0.12	< 0.001
Mean |bias| (%)	3.14[c,f]	13.52[b,f,g,h]	12.27[a,c,d,e]	24.58[+]	2.98[a,b]	3.96[e,h]	3.28[d,g]	< 0.001
Precision (%)	5.29[d,g,i]	10.94[b,g,h]	17.06[a,d,e,f]	14.51[c,i,j,k]	3.87[a,b,c]	4.51[f,k]	7.17[e,h,j]	< 0.001
CheckMe™ O2+								
N	30	31	31	31	32	30	31	-
RMSE (%, 95% CI)	2.37(1.41, 3.27)	17.52(14.39, 20.44)	18.12(13.91, 22.0)	20.11(13.32, 25.96)	7.3(4.89, 9.61)	11.98(8.75, 15.08)	10.65(7.55, 13.52)	-
Mean bias (%)	-0.13b	-13.27[+]	5.26	11.97[a,b,c,d]	0.67[a]	0.1[d]	2.02[c]	< 0.001
Mean |bias| (%)	1.57[d,e,g,h]	14.66[b,e,f]	14.06[a,d]	13.84[c,g]	5.17[a,b,c]	9.17$_{h}$	7.69[f]	< 0.001
Precision (%)	2.43[c,e,f,g]	10.26[e]	15.97[a,c,d]	13.17[b,f]	6.28[a,b]	11.62$_{g}$	9.72[d]	< 0.001
Philips MX450								
N	33	32	33	33	32	30	31	-
RMSE (%, 95% CI)	2.42(1.58, 3.19)	10.73(3.98, 16.71)	27.65(15.92, 37.84)	10.13(2.66, 15.82)	2.81(1.8, 3.76)	19.4(5.38, 30.91)	2.77(1.83, 3.73)	-
Mean bias (%)	-0.73	-1.78	6.5	2.82	-0.38	4.55	-1.05	0.119
Mean |bias| (%)	1.61[c]	5.16[b]	15.2[a,b,c,d,e]	4.52[e]	1.88[a]	8.18	1.89[d]	< 0.001
Precision (%)	2.38[c]	10.77[b]	27.7[a,b,c,d,e]	10.03[e]	2.86[a]	18.32	2.56[d]	< 0.001
Wavelet								
N	18	2	4	8	7	5	5	-
RMSE (%, 95% CI)	5.04(2.86, 6.92)	2.5(2.48, 2.52)	11.18(0.5, 19.31)	20.68(4.47, 29.97)	6.4(3.5, 9.23)	6.59(4.6, 7.79)	3.5(2.05, 4.98)	-
Mean bias (%)	-2.72	2.5	-5.97	10.35	-0.15	-3.49	2.14	0.052
Mean |bias| (%)	3.45	2.5	6.22	13.27	5.36	6.19	3.13	0.184
Precision (%)	3.93	-	13.33	16.15	7.48	7.15	3.5	0.136
WristOx2® 3150								
N	32	32	29	30	32	24	32	-
RMSE (%, 95% CI)	1.94(0.84, 2.82)	11.77(7.21, 15.56)	10.24(4.88, 14.51)	11.7(4.41, 17.48)	2.6(1.76, 3.36)	14.9(5.83, 22.6)	3.27(1.85, 4.53)	-
Mean bias (%)	-0.48	-3.06[b]	-2.81[a]	4.25[a,b]	0.14	-0.12	-0.97	0.045
Mean |bias| (%)	1.05[b]	6.72	6.05	6.22	1.86[a]	8.29[a,b,c]	2.06[c]	< 0.001
Precision (%)	1.85[b]	10.98	8.92	11.21	2.49[a]	14.03[a,b,c]	2.94[c]	< 0.001

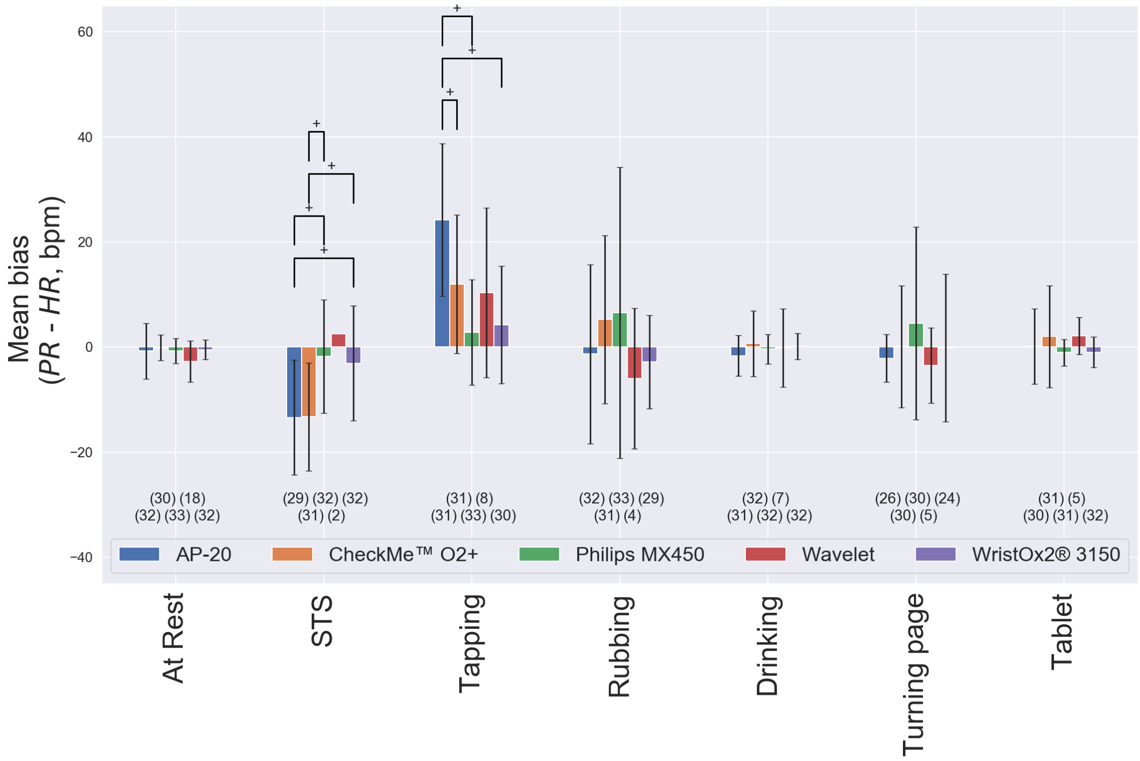

Figure A.1 – Comparison of the mean bias and precision between devices, for each movement type. The number of points available per device is shown below each bar (the number of HR target windows for each activity is presented in Table A.1). + Different from each other.

PR estimation in the hypoxia exposure phase

Table A.2 compares the agreement between the PR and HR estimates for each device during the hypoxia exposure phase. Both the Philips MX450 and the WristOx2® 3150 presented a significantly lower RMSE than the remaining devices. On the other hand, the Wavelet presented a higher number of dropouts, higher mean bias and higher variance (measured via the precision metric). All devices were within the acceptable accuracy limit (i.e. RMSE ≤ 5%).

Table A.2 – Comparison of the accuracy (RMSE) and the mean bias between devices during the hypoxia exposure phase. There was a total of 215 HR target windows in this phase. N – number of available test PR points. +Different from other values. a,b Different from each other.

	Philips MX 450	*CheckMe™ O2+*	*WristOx2® 3150*	*Wavelet*	*AP-20*	*P*
N	215	207	209	157	214	-
RMSE (% ,95% CI)	1.67 (1.43, 1.92)	2.89 (2.08, 3.65)	1.49 (1.19, 1.82)	4.87 (3.49, 6.17)	3.18 (2.5, 3.9)	-
Mean bias (%)	-0.92[a]	-0.02[a]	-0.22	-0.22	-0.42	0.021
Mean |bias|(%)	1.21[b]	1.42	0.88[a]	2.75[+]	1.89[a,b]	< 0.001
Precision (%)	1.38[b]	2.88	1.47[a]	4.88[+]	3.16[a,b]	< 0.001

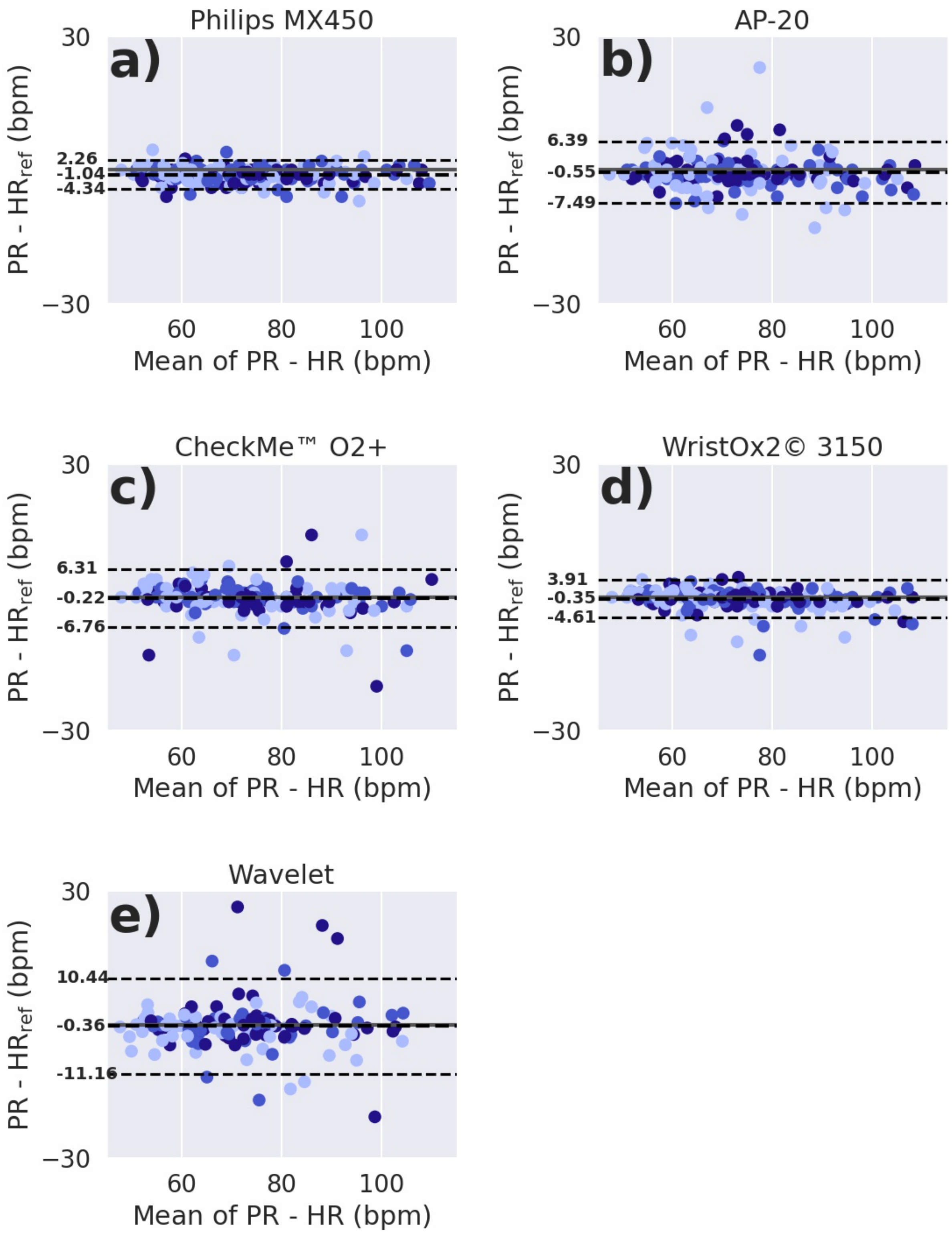

Figure A.2 – a-e) Bland-Altman plots for the Philips MX450, AP-20, CheckMe^{TM} O2+, WristOx2$^{©}$ 3150 and Wavelet PR estimation, respectively. The mean bias and limits of agreement are shown in dashed lines.

Discussion

In this sub-study, the performance of PR estimation in wearable pulse oximeters was analysed. In the movement phase it was observed that while the Wavelet device presented PR estimates comparable to the other devices when at rest (18 estimates out of 33 targets), it presented a much lower number of estimates on the remainder tasks (between 2 and 8), and therefore its results may not be comparable with the remainder devices. Amongst the latter, the STS and tapping tasks were the most challenging for PR estimation, especially to the AP-20 and the CheckMe^{TM} O2+, both showing a significantly higher mean bias in these tasks than the remaining finger-worn devices.

In the hypoxia exposure phase the Wavelet also presented a higher number of dropouts, mean absolute bias and variance (measured via the precision metric), but its accuracy was still within the acceptable limit. We note the patients were at rest during this phase and the Wavelet was able to compute PR for 157 of the total of 215 target HR windows, in contrast to the 32 SpO_2 values that it was able to compute for the same data windows, as discussed for the SpO_2 dataset (see main manuscript). I.e., although there were Wavelet data available during the hypoxia exposure phase target SpO_2 windows, their algorithm design was only able to compute PR (usually from the infrared light), but not SpO_2, which also requires the red-light signal with high signal to noise ratio. As discussed, this device does not acquire waveform data continuously and it is possible that for some of the remaining 62 target HR windows there were no data to compute the PR, making its comparison with the continuous finger-worn pulse oximeters estimates challenging.

Our findings show that the differences between the finger- worn pulse oximeters algorithms also result in different biases in PR estimation, especially when challenged by motion. The AP-20 in particular, which showed good performance in SpO_2 estimation, presented a lower performance in PR estimation amongst the selected finger-worn devices. When considering the results from both phases, the WristOx2® 3150 showed to have a PR estimate performance most comparable to that of the standard care Philips MX450 monitor pulse oximeter.

Limitations

The sample size for this study was calculated based on the ISO 80601-2-61:2019 guidelines to evaluate oximeter's performance in detecting changes in SpO_2. Therefore, the dataset might not have statistical power to identify differences in the PR estimation between oximeters, or between different motion tasks. We note also that in this dataset the HR changed in the range between 50 and 110 bpm, a subset of the range used in the guideline to validate heart rate monitors, ANSI/AAMI EC13:2002, between 20 and 200 bpm. Finally, the results are based on limited demographics and therefore do not generalise for the wider population (e.g., darker skin types, or patients with comorbidities).

Conclusions

Our sub-study showed that wearable, wireless transmission-mode, pulse oximeters presented differences in the PR estimation accuracy, especially when challenged by motion. PR estimation performance should also be considered when selecting wearable devices to use in a clinical setting. All devices estimated PR within the acceptable clinical range (at rest). The WristOx2® 3150 PR estimation was the most comparable to that of the standard monitor.
