# Supplementary material for: The Use of Wearable Pulse Oximeters in the Prompt Detection of Hypoxemia and During Movement: Diagnostic Accuracy Study
Source: J Med Internet Res. 2022 Feb 15;24(2):e28890. doi: 10.2196/28890 (PMC8889481; doi:10.2196/28890)
Supplement: Multimedia Appendix 2 [file jmir_v24i2e28890_app2.pdf]

Appendix B

Results of Wavelet's SpO_2 estimation

Participants

The demographics matched those analysed in the main manuscript results section (from 33 participants).

SpO_2 estimation in the movement phase

The performance metrics values for each device are shown in Table B.1. We note that very few estimates were provided by the Wavelet, which only presented four points at rest, and less than that for the other motion tasks. Therefore, its SpO_2 estimation accuracy was deemed not comparable to the other devices for the movement phase.

SpO2 estimation in the hypoxia exposure phase

Table B.2 shows the performance of the Wavelet device across the range of SaO_2 targets of the hypoxia exposure phase. The Wavelet had a significantly lower performance than that of the other devices, with a mean bias and RMSE that were about 8 and 3 times higher than the other devices, respectively. SaO_2 subgroup analysis (Table B.3, Figure B.1B and Figure B.2, the latter including all devices for comparison) also showed that the Wavelet achieved a significantly lower performance across all subgroups, most notably for lower SaO_2 ranges. This device showed a high number of dropouts, i.e. only 12, 13, 14 estimates were available from a total of 60, 74, and 79 SaO_2 target windows in the severe-hypoxia, mild-hypoxia and normoxia ranges, respectively.

Sensitivity and Specificity

From a total of 215 SaO_2 targets, while 202 had complete data for all the devices (Table 5 in the main manuscript), the Wavelet only presented 39 data points. The latter was again not comparable to the finger-worn pulse oximeters and due to the low amount of estimates it was not possible to compute these metrics.

Discussion

We found that the performance of the Wavelet's (wrist-only) SpO_2 estimation was not comparable to that of the finger-based oximeters. We note that its mode of operation is different to that of the finger-worn probes: for most of the 2-minute windows there are only SpO_2 estimates (at 1 Hz) for 50% of the time. It is important to note that the ISO 9919:2005 standards for pulse oximeters require data to be updated at least every 30 seconds, but the Wavelet's configuration available to our study was limited to selecting 2-minute SpO_2 test windows. Other differences include the fact that the photoplethysmography waveform is obtained by light reflectance, rather than light transmission as with conventional pulse oximetry, and that the SpO_2 estimation and signal quality algorithms run retrospectively in Wavelet Health's cloud platform (once the waveforms are received). This technology is experimental for SpO_2 estimation, i.e. did not yet receive regulatory approved, and the results confirm that very few SpO_2 estimates were computed during the motion tasks (only four estimates out of 33 experiments while at rest and no estimates for three of the motion tasks). Additionally, during the hypoxia phase, Wavelet had waveform data for at least 157 of the total of 215 target SaO_2 windows, but only estimated SpO_2 for 39 targets (this is further discussed in the Pulse Rate estimation accuracy analysis, Appendix B). Finally, the device was not capable of providing estimates for SaO_2

values below 90% (overall mean bias was 8.48 %, Table B.2). It was thus not possible to compute the primary outcome for this device in this study.

Limitations

At the time of the study the Wavelet had not yet acquired regulatory approval, and so the device was not expected to perform like the remainder.

Conclusions

The wrist-worn Wavelet device was not able to detect hypoxaemia. This device is in the early stages of development for its SpO_2 estimation algorithm and is too experimental for use in clinical settings. It should be noted that new wrist-worn reflectance-mode pulse oximeters have become available since we completed our clinical study.

Table B.1 – Comparison of the accuracy and the mean bias of Wavelet's SpO_2 estimation between different motion tasks, for each device, for 33 participants. One-way ANOVA followed by the Tukey-Kramer test was used to evaluate differences in the mean bias and mean absolute bias between tasks. Levene's test was used in the case of the precision. N – number of available SpO_2 points.

	At Rest	***STS***	***Rubbing***	***Tapping***	***Drinking***	***Turning page***	***Tablet***	***P***
Wavelet								
N	4	0	2	0	1	0	3	-
RMSE (%, 95% CI)	2.02 (1.14, 2.91)	-	0.96 (0.8, 1.1)	-	1.2 (1.2, 1.2)	-	1.47 (1.0, 1.8)	-
Mean bias (%)	1.17	-	0.95	-	1.2	-	1.43	0.983
Mean |bias| (%)	1.81	-	0.95	-	1.2	-	1.43	0.631
Precision (%)	0.81	-	-	-	-	-	-	-

Table B.2 – Comparison of the accuracy and the mean bias of the SpO_2 estimation between devices, during the hypoxia exposure phase. There was a total of 215 SaO_2 target windows in this phase. N – number of available SpO_2 points. [+] Different from other values. [a] Different from each other.

	Philips MX 450	*CheckMe™ O2+*	*WristOx2® 3150*	*Wavelet*	*AP20*	*P*
N	215	207	209	39	214	-
RMSE (%, 95% CI)	2.67 (2.31, 3.06)	3.20 (2.85, 3.56)	3.33 (2.85, 3.86)	9.77 (8.37, 11.1)	2.86 (2.44, 3.25)	-
Mean bias (%)	0.49[a]	-0.22	-1.92[+]	8.48[+]	-0.3[a]	< 0.001
Mean |bias| (%)	1.92	2.42	2.40	8.54[+]	2.00	< 0.001
Precision (%)	2.62[a]	3.16[a]	2.73	1.90[+]	2.83	< 0.001

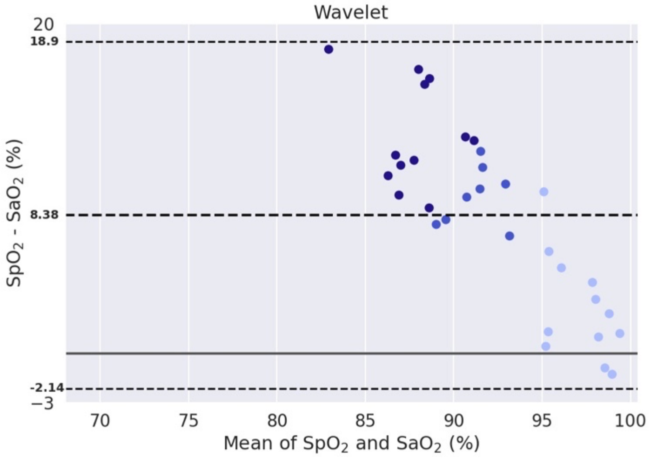

Figure B.1 – a) Bland-Altman plot for the Wavelet's SpO_2 estimates. The Wavelet was not able to compute low SpO_2.

Table B.3 - Comparison of the accuracy and the mean bias of Wavelet's SpO_2 estimation between three SaO_2 subgroups. The three SaO_2 subgroups are: severe-hypoxia (SaO_2 below 85%), mild-hypoxia (SaO_2 between 85% and 89%) and normoxia (SaO_2 at or above 90%). N - number of available SpO_2 points.

	< 85%	***85-89%***	***90-100%***	***P[a]***
Wavelet				
N	12	13	14	-
RMSE (%, 95% CI)	13.6 (11.84, 15.32)	9.65 (8.55, 10.67)	4.57 (2.91, 6.14)	-
Mean bias (%)	13.25[+]	9.45[+]	3.48[+]	< 0.001
Mean |bias| (%)	13.25[+]	9.45[+]	3.66[+]	< 0.001
Precision (%)	2.39	1.92	1.53	0.322
Total ABGs (N)	60	76	79	-

[a]For each device, one-way ANOVA followed by the Tukey's test was used to evaluate differences in the mean bias and mean absolute bias between subgroups. Levene's test was used in the case of the precision. [+]Different from all values.

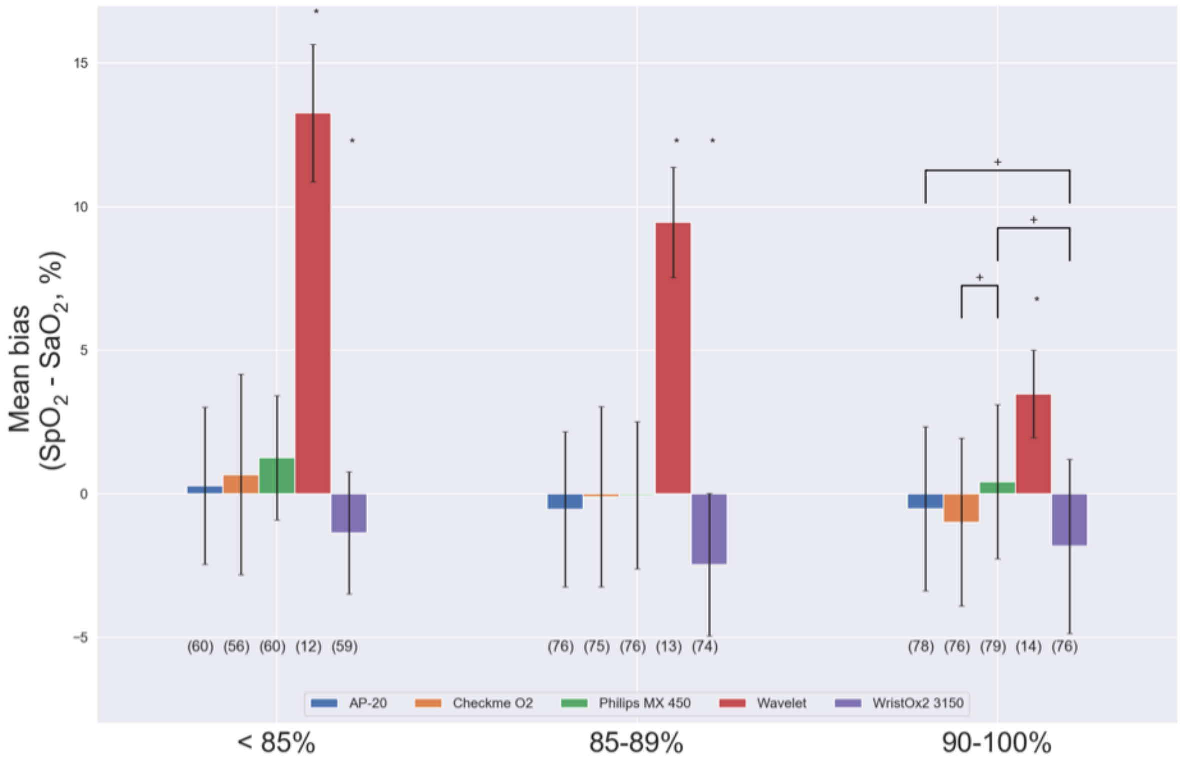

Figure B.2 – Comparison of the mean bias and precision between devices for the three SaO_2 subgroups: severe-hypoxia (SaO_2 lower than 85%), mild-hypoxia (SaO_2 between 85% and 89%) and normoxia (SaO_2 equal or greater than 90%). The number of points available per device is presented below each bar (in a total of 215 SaO_2 target windows). For each subgroup, one-way ANOVA followed by the Tukey's test was used to evaluate differences in the mean bias between devices. * Different from other values. $^+$ Different from each other.
